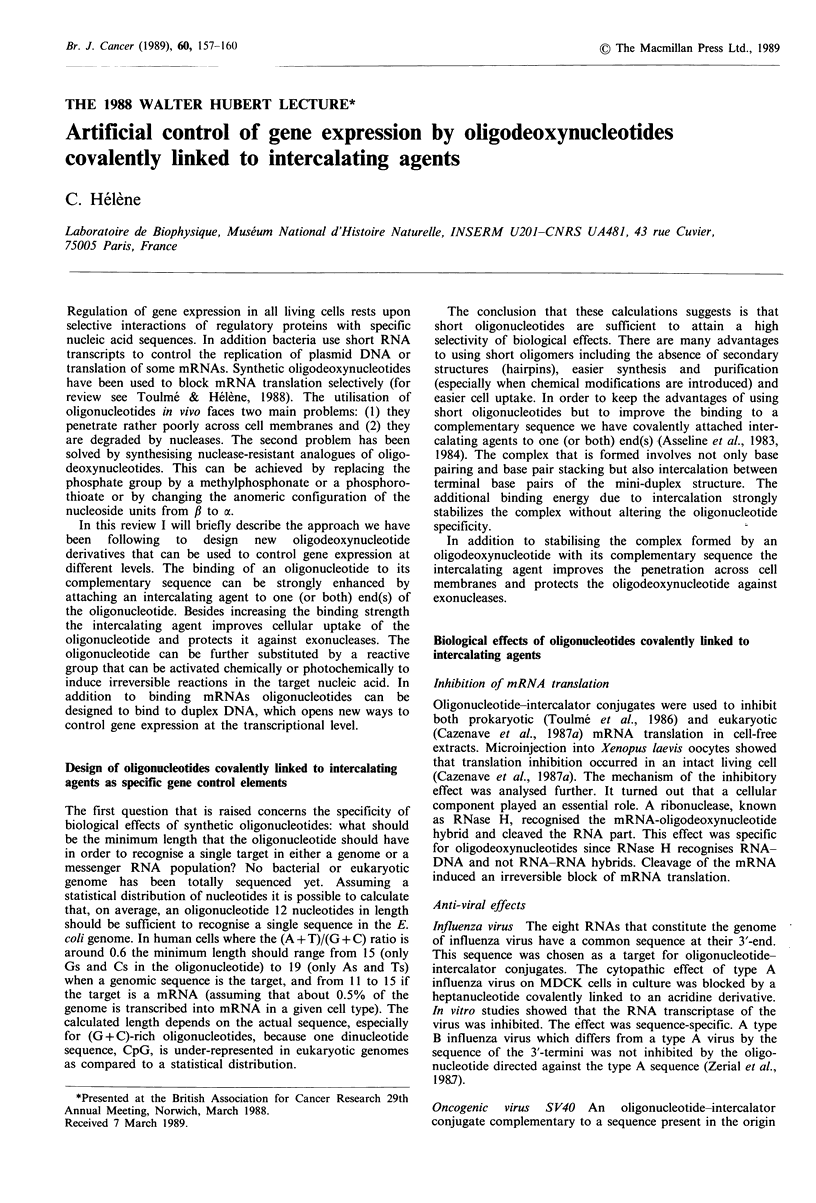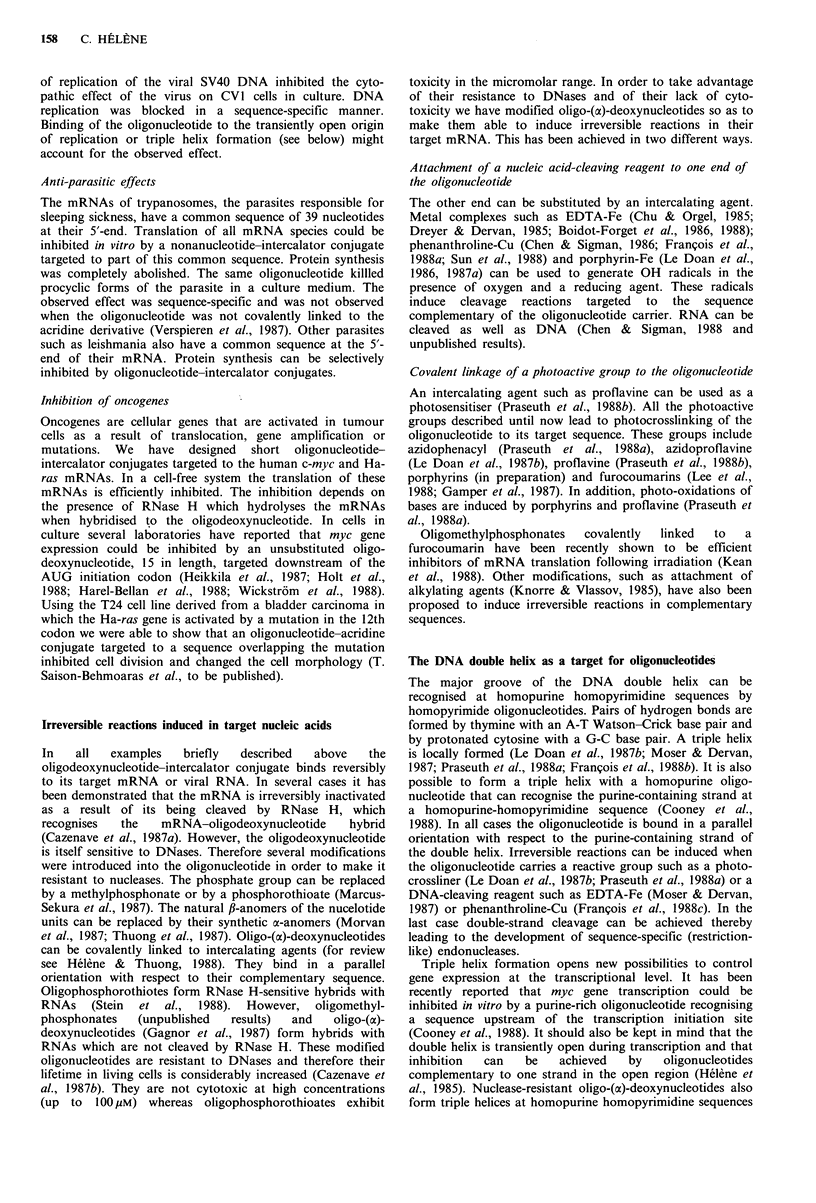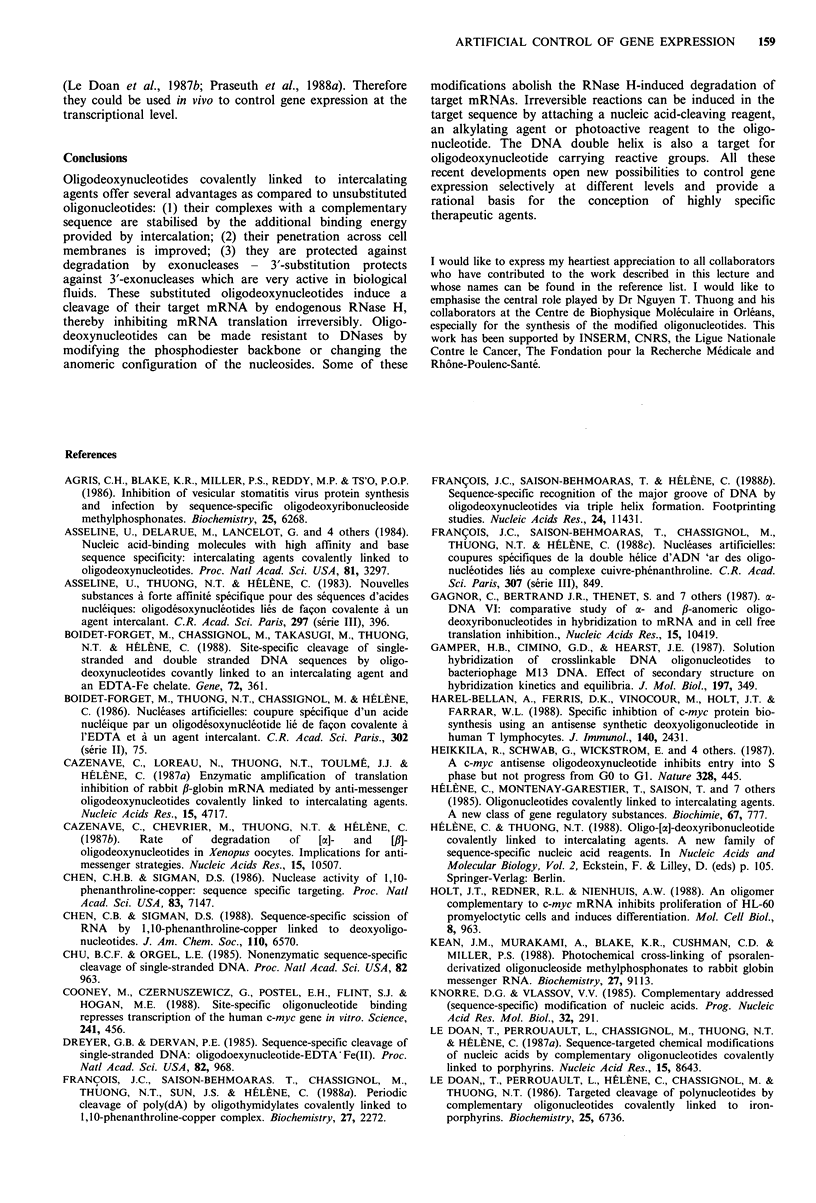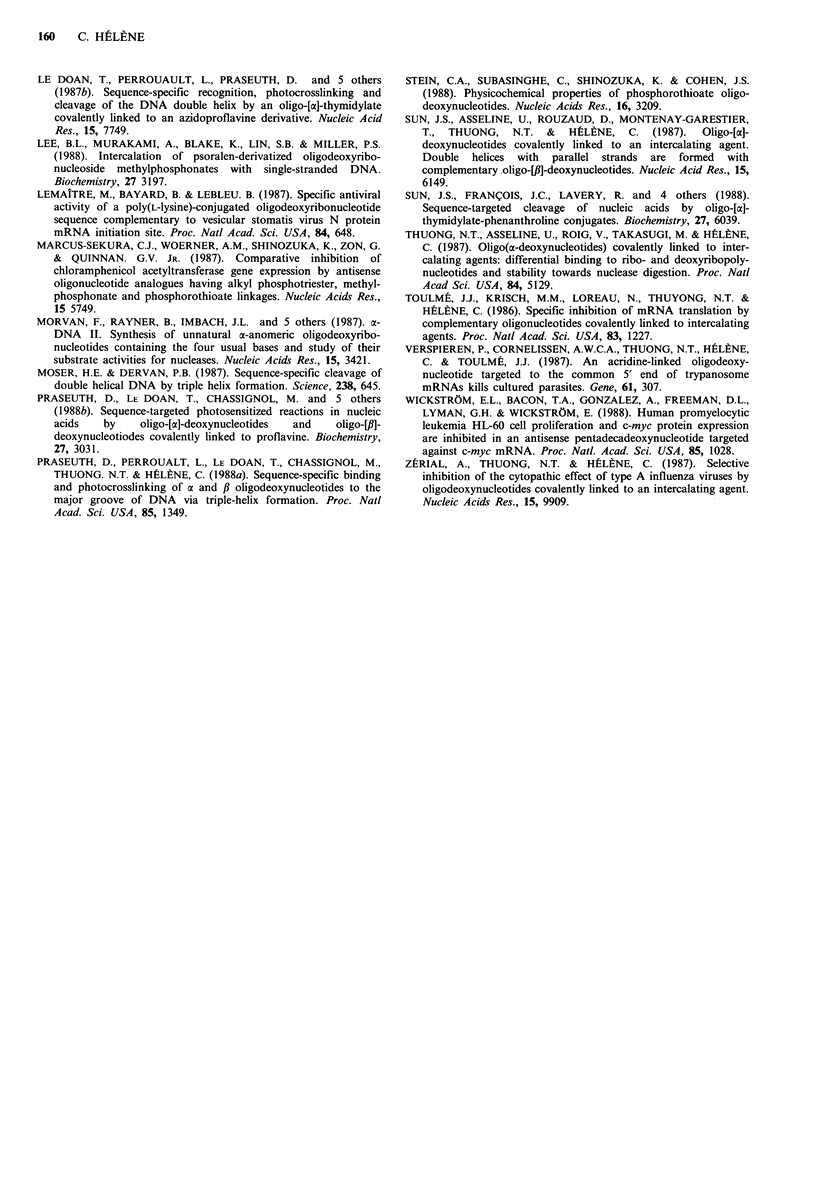# The 1988 Walter Hubert lecture. Artificial control of gene expression by oligodeoxynucleotides covalently linked to intercalating agents.

**DOI:** 10.1038/bjc.1989.242

**Published:** 1989-08

**Authors:** C. HÃ©lÃ¨ne

**Affiliations:** Laboratoire de Biophysique, INSERM U201-CNRS UA481, Paris, France.


Br. J. Cancer (1989), **60**, 157–160 © The Macmillan Press Ltd., 1989

**THE 1988 WALTER HUBERT LECTURE***

### Artificial control of gene expression by oligodeoxynucleotides covalently linked to intercalating agents

C. Hélène

*Laboratoire de Biophysique, Muséum National d'Histoire Naturelle, INSERM U201–CNRS UA481, 43 rue Cuvier, 75005 Paris, France*

Regulation of gene expression in all living cells rests upon selective interactions of regulatory proteins with specific nucleic acid sequences. In addition bacteria use short RNA transcripts to control the replication of plasmid DNA or translation of some mRNAs. Synthetic oligodeoxynucleotides have been used to block mRNA translation selectively (for review see Toulmé & Hélène, 1988). The utilisation of oligonucleotides *in vivo* faces two main problems: (1) they penetrate rather poorly across cell membranes and (2) they are degraded by nucleases. The second problem has been solved by synthesising nuclease-resistant analogues of oligodeoxynucleotides. This can be achieved by replacing the phosphate group by a methylphosphonate or a phosphorothioate or by changing the anomeric configuration of the nucleoside units from $\beta$ to $\alpha$.

In this review I will briefly describe the approach we have been following to design new oligodeoxynucleotide derivatives that can be used to control gene expression at different levels. The binding of an oligonucleotide to its complementary sequence can be strongly enhanced by attaching an intercalating agent to one (or both) end(s) of the oligonucleotide. Besides increasing the binding strength the intercalating agent improves cellular uptake of the oligonucleotide and protects it against exonucleases. The oligonucleotide can be further substituted by a reactive group that can be activated chemically or photochemically to induce irreversible reactions in the target nucleic acid. In addition to binding mRNAs oligonucleotides can be designed to bind to duplex DNA, which opens new ways to control gene expression at the transcriptional level.

### Design of oligonucleotides covalently linked to intercalating agents as specific gene control elements

The first question that is raised concerns the specificity of biological effects of synthetic oligonucleotides: what should be the minimum length that the oligonucleotide should have in order to recognise a single target in either a genome or a messenger RNA population? No bacterial or eukaryotic genome has been totally sequenced yet. Assuming a statistical distribution of nucleotides it is possible to calculate that, on average, an oligonucleotide 12 nucleotides in length should be sufficient to recognise a single sequence in the *E. coli* genome. In human cells where the (A+T)/(G+C) ratio is around 0.6 the minimum length should range from 15 (only Gs and Cs in the oligonucleotide) to 19 (only As and Ts) when a genomic sequence is the target, and from 11 to 15 if the target is a mRNA (assuming that about 0.5% of the genome is transcribed into mRNA in a given cell type). The calculated length depends on the actual sequence, especially for (G+C)-rich oligonucleotides, because one dinucleotide sequence, CpG, is under-represented in eukaryotic genomes as compared to a statistical distribution.

The conclusion that these calculations suggests is that short oligonucleotides are sufficient to attain a high selectivity of biological effects. There are many advantages to using short oligomers including the absence of secondary structures (hairpins), easier synthesis and purification (especially when chemical modifications are introduced) and easier cell uptake. In order to keep the advantages of using short oligonucleotides but to improve the binding to a complementary sequence we have covalently attached intercalating agents to one (or both) end(s) (Asseline *et al.*, 1983, 1984). The complex that is formed involves not only base pairing and base pair stacking but also intercalation between terminal base pairs of the mini-duplex structure. The additional binding energy due to intercalation strongly stabilizes the complex without altering the oligonucleotide specificity.

In addition to stabilising the complex formed by an oligodeoxynucleotide with its complementary sequence the intercalating agent improves the penetration across cell membranes and protects the oligodeoxynucleotide against exonucleases.

### Biological effects of oligonucleotides covalently linked to intercalating agents

### *Inhibition of mRNA translation*

Oligonucleotide–intercalator conjugates were used to inhibit both prokaryotic (Toulmé *et al.*, 1986) and eukaryotic (Cazenave *et al.*, 1987*a*) mRNA translation in cell-free extracts. Microinjection into *Xenopus laevis* oocytes showed that translation inhibition occurred in an intact living cell (Cazenave *et al.*, 1987*a*). The mechanism of the inhibitory effect was analysed further. It turned out that a cellular component played an essential role. A ribonuclease, known as RNase H, recognised the mRNA-oligodeoxynucleotide hybrid and cleaved the RNA part. This effect was specific for oligodeoxynucleotides since RNase H recognises RNA–DNA and not RNA–RNA hybrids. Cleavage of the mRNA induced an irreversible block of mRNA translation.

### *Anti-viral effects*

*Influenza virus* The eight RNAs that constitute the genome of influenza virus have a common sequence at their 3′-end. This sequence was chosen as a target for oligonucleotide–intercalator conjugates. The cytopathic effect of type A influenza virus on MDCK cells in culture was blocked by a heptanucleotide covalently linked to an acridine derivative. *In vitro* studies showed that the RNA transcriptase of the virus was inhibited. The effect was sequence-specific. A type B influenza virus which differs from a type A virus by the sequence of the 3′-termini was not inhibited by the oligonucleotide directed against the type A sequence (Zerial *et al.*, 1987).

*Oncogenic virus SV40* An oligonucleotide–intercalator conjugate complementary to a sequence present in the origin

*Presented at the British Association for Cancer Research 29th Annual Meeting, Norwich, March 1988.
Received 7 March 1989.

of replication of the viral SV40 DNA inhibited the cytopathic effect of the virus on CV1 cells in culture. DNA replication was blocked in a sequence-specific manner. Binding of the oligonucleotide to the transiently open origin of replication or triple helix formation (see below) might account for the observed effect.

*Anti-parasitic effects*

The mRNAs of trypanosomes, the parasites responsible for sleeping sickness, have a common sequence of 39 nucleotides at their 5′-end. Translation of all mRNA species could be inhibited *in vitro* by a nonanucleotide–intercalator conjugate targeted to part of this common sequence. Protein synthesis was completely abolished. The same oligonucleotide killled procyclic forms of the parasite in a culture medium. The observed effect was sequence-specific and was not observed when the oligonucleotide was not covalently linked to the acridine derivative (Verspieren *et al.*, 1987). Other parasites such as leishmania also have a common sequence at the 5′-end of their mRNA. Protein synthesis can be selectively inhibited by oligonucleotide–intercalator conjugates.

*Inhibition of oncogenes*

Oncogenes are cellular genes that are activated in tumour cells as a result of translocation, gene amplification or mutations. We have designed short oligonucleotide–intercalator conjugates targeted to the human c-*myc* and Ha-*ras* mRNAs. In a cell-free system the translation of these mRNAs is efficiently inhibited. The inhibition depends on the presence of RNase H which hydrolyses the mRNAs when hybridised to the oligodeoxynucleotide. In cells in culture several laboratories have reported that *myc* gene expression could be inhibited by an unsubstituted oligodeoxynucleotide, 15 in length, targeted downstream of the AUG initiation codon (Heikkila *et al.*, 1987; Holt *et al.*, 1988; Harel-Bellan *et al.*, 1988; Wickström *et al.*, 1988). Using the T24 cell line derived from a bladder carcinoma in which the Ha-*ras* gene is activated by a mutation in the 12th codon we were able to show that an oligonucleotide–acridine conjugate targeted to a sequence overlapping the mutation inhibited cell division and changed the cell morphology (T. Saison-Behmoaras *et al.*, to be published).

### Irreversible reactions induced in target nucleic acids

In all examples briefly described above the oligodeoxynucleotide–intercalator conjugate binds reversibly to its target mRNA or viral RNA. In several cases it has been demonstrated that the mRNA is irreversibly inactivated as a result of its being cleaved by RNase H, which recognises the mRNA–oligodeoxynucleotide hybrid (Cazenave *et al.*, 1987*a*). However, the oligodeoxynucleotide is itself sensitive to DNases. Therefore several modifications were introduced into the oligonucleotide in order to make it resistant to nucleases. The phosphate group can be replaced by a methylphosphonate or by a phosphorothioate (Marcus-Sekura *et al.*, 1987). The natural $\beta$-anomers of the nucelotide units can be replaced by their synthetic $\alpha$-anomers (Morvan *et al.*, 1987; Thuong *et al.*, 1987). Oligo-($\alpha$)-deoxynucleotides can be covalently linked to intercalating agents (for review see Hélène & Thuong, 1988). They bind in a parallel orientation with respect to their complementary sequence. Oligophosphorothiotes form RNase H-sensitive hybrids with RNAs (Stein *et al.*, 1988). However, oligomethylphosphonates (unpublished results) and oligo-($\alpha$)-deoxynucleotides (Gagnor *et al.*, 1987) form hybrids with RNAs which are not cleaved by RNase H. These modified oligonucleotides are resistant to DNases and therefore their lifetime in living cells is considerably increased (Cazenave *et al.*, 1987*b*). They are not cytotoxic at high concentrations (up to 100 $\mu$M) whereas oligophosphorothioates exhibit toxicity in the micromolar range. In order to take advantage of their resistance to DNases and of their lack of cytotoxicity we have modified oligo-($\alpha$)-deoxynucleotides so as to make them able to induce irreversible reactions in their target mRNA. This has been achieved in two different ways.

*Attachment of a nucleic acid-cleaving reagent to one end of the oligonucleotide*

The other end can be substituted by an intercalating agent. Metal complexes such as EDTA-Fe (Chu & Orgel, 1985; Dreyer & Dervan, 1985; Boidot-Forget *et al.*, 1986, 1988); phenanthroline-Cu (Chen & Sigman, 1986; François *et al.*, 1988*a*; Sun *et al.*, 1988) and porphyrin-Fe (Le Doan *et al.*, 1986, 1987*a*) can be used to generate OH radicals in the presence of oxygen and a reducing agent. These radicals induce cleavage reactions targeted to the sequence complementary of the oligonucleotide carrier. RNA can be cleaved as well as DNA (Chen & Sigman, 1988 and unpublished results).

*Covalent linkage of a photoactive group to the oligonucleotide*

An intercalating agent such as proflavine can be used as a photosensitiser (Praseuth *et al.*, 1988*b*). All the photoactive groups described until now lead to photocrosslinking of the oligonucleotide to its target sequence. These groups include azidophenacyl (Praseuth *et al.*, 1988*a*), azidoproflavine (Le Doan *et al.*, 1987*b*), proflavine (Praseuth *et al.*, 1988*b*), porphyrins (in preparation) and furocoumarins (Lee *et al.*, 1988; Gamper *et al.*, 1987). In addition, photo-oxidations of bases are induced by porphyrins and proflavine (Praseuth *et al.*, 1988*a*).

Oligomethylphosphonates covalently linked to a furocoumarin have been recently shown to be efficient inhibitors of mRNA translation following irradiation (Kean *et al.*, 1988). Other modifications, such as attachment of alkylating agents (Knorre & Vlassov, 1985), have also been proposed to induce irreversible reactions in complementary sequences.

### The DNA double helix as a target for oligonucleotides

The major groove of the DNA double helix can be recognised at homopurine homopyrimidine sequences by homopyrimide oligonucleotides. Pairs of hydrogen bonds are formed by thymine with an A-T Watson–Crick base pair and by protonated cytosine with a G-C base pair. A triple helix is locally formed (Le Doan *et al.*, 1987*b*; Moser & Dervan, 1987; Praseuth *et al.*, 1988*a*; François *et al.*, 1988*b*). It is also possible to form a triple helix with a homopurine oligonucleotide that can recognise the purine-containing strand at a homopurine-homopyrimidine sequence (Cooney *et al.*, 1988). In all cases the oligonucleotide is bound in a parallel orientation with respect to the purine-containing strand of the double helix. Irreversible reactions can be induced when the oligonucleotide carries a reactive group such as a photocrossliner (Le Doan *et al.*, 1987*b*; Praseuth *et al.*, 1988*a*) or a DNA-cleaving reagent such as EDTA-Fe (Moser & Dervan, 1987) or phenanthroline-Cu (François *et al.*, 1988*c*). In the last case double-strand cleavage can be achieved thereby leading to the development of sequence-specific (restriction-like) endonucleases.

Triple helix formation opens new possibilities to control gene expression at the transcriptional level. It has been recently reported that *myc* gene transcription could be inhibited *in vitro* by a purine-rich oligonucleotide recognising a sequence upstream of the transcription initiation site (Cooney *et al.*, 1988). It should also be kept in mind that the double helix is transiently open during transcription and that inhibition can be achieved by oligonucleotides complementary to one strand in the open region (Hélène *et al.*, 1985). Nuclease-resistant oligo-($\alpha$)-deoxynucleotides also form triple helices at homopurine homopyrimidine sequences

(Le Doan *et al.*, 1987*b*; Praseuth *et al.*, 1988*a*). Therefore they could be used *in vivo* to control gene expression at the transcriptional level.

### Conclusions

Oligodeoxynucleotides covalently linked to intercalating agents offer several advantages as compared to unsubstituted oligonucleotides: (1) their complexes with a complementary sequence are stabilised by the additional binding energy provided by intercalation; (2) their penetration across cell membranes is improved; (3) they are protected against degradation by exonucleases – 3′-substitution protects against 3′-exonucleases which are very active in biological fluids. These substituted oligodeoxynucleotides induce a cleavage of their target mRNA by endogenous RNase H, thereby inhibiting mRNA translation irreversibly. Oligodeoxynucleotides can be made resistant to DNases by modifying the phosphodiester backbone or changing the anomeric configuration of the nucleosides. Some of these modifications abolish the RNase H-induced degradation of target mRNAs. Irreversible reactions can be induced in the target sequence by attaching a nucleic acid-cleaving reagent, an alkylating agent or photoactive reagent to the oligonucleotide. The DNA double helix is also a target for oligodeoxynucleotide carrying reactive groups. All these recent developments open new possibilities to control gene expression selectively at different levels and provide a rational basis for the conception of highly specific therapeutic agents.

I would like to express my heartiest appreciation to all collaborators who have contributed to the work described in this lecture and whose names can be found in the reference list. I would like to emphasise the central role played by Dr Nguyen T. Thuong and his collaborators at the Centre de Biophysique Moléculaire in Orléans, especially for the synthesis of the modified oligonucleotides. This work has been supported by INSERM, CNRS, the Ligue Nationale Contre le Cancer, The Fondation pour la Recherche Médicale and Rhône-Poulenc-Santé.

### References

AGRIS, C.H., BLAKE, K.R., MILLER, P.S., REDDY, M.P. & TS'O, P.O.P. (1986). Inhibition of vesicular stomatitis virus protein synthesis and infection by sequence-specific oligodeoxyribonucleoside methylphosphonates. *Biochemistry*, **25**, 6268.

ASSELINE, U., DELARUE, M., LANCELOT, G. and 4 others (1984). Nucleic acid-binding molecules with high affinity and base sequence specificity: intercalating agents covalently linked to oligodeoxynucleotides. *Proc. Natl Acad. Sci. USA*, **81**, 3297.

ASSELINE, U., THUONG, N.T. & HÉLÈNE, C. (1983). Nouvelles substances à forte affinité spécifique pour des séquences d'acides nucléiques: oligodésoxynucléotides liés de façon covalente à un agent intercalant. *C.R. Acad. Sci. Paris*, **297** (série III), 396.

BOIDET-FORGET, M., CHASSIGNOL, M., TAKASUGI, M., THUONG, N.T. & HÉLÈNE, C. (1988). Site-specific cleavage of single-stranded and double stranded DNA sequences by oligodeoxynucleotides covantly linked to an intercalating agent and an EDTA-Fe chelate. *Gene*, **72**, 361.

BOIDET-FORGET, M., THUONG, N.T., CHASSIGNOL, M. & HÉLÈNE, C. (1986). Nucléases artificielles: coupure spécifique d'un acide nucléique par un oligodésoxynucléotide lié de façon covalente à l'EDTA et à un agent intercalant. *C.R. Acad. Sci. Paris.*, **302** (série II), 75.

CAZENAVE, C., LOREAU, N., THUONG, N.T., TOULMÉ, J.J. & HÉLÈNE, C. (1987*a*) Enzymatic amplification of translation inhibition of rabbit $\beta$-globin mRNA mediated by anti-messenger oligodeoxynucleotides covalently linked to intercalating agents. *Nucleic Acids Res.*, **15**, 4717.

CAZENAVE, C., CHEVRIER, M., THUONG, N.T. & HÉLÈNE, C. (1987*b*). Rate of degradation of [$\alpha$]- and [$\beta$]-oligodeoxynucleotides in *Xenopus* oocytes. Implications for anti-messenger strategies. *Nucleic Acids Res.*, **15**, 10507.

CHEN, C.H.B. & SIGMAN, D.S. (1986). Nuclease activity of 1,10-phenanthroline-copper: sequence specific targeting. *Proc. Natl Acad. Sci. USA*, **83**, 7147.

CHEN, C.B. & SIGMAN, D.S. (1988). Sequence-specific scission of RNA by 1,10-phenanthroline-copper linked to deoxyoligonucleotides. *J. Am. Chem. Soc.*, **110**, 6570.

CHU, B.C.F. & ORGEL, L.E. (1985). Nonenzymatic sequence-specific cleavage of single-stranded DNA. *Proc. Natl Acad. Sci. USA*, **82** 963.

COONEY, M., CZERNUSZEWICZ, G., POSTEL, E.H., FLINT, S.J. & HOGAN, M.E. (1988). Site-specific oligonucleotide binding represses transcription of the human c-*myc* gene *in vitro*. *Science*, **241**, 456.

DREYER, G.B. & DERVAN, P.E. (1985). Sequence-specific cleavage of single-stranded DNA: oligodoexynucleotide-EDTA·Fe(II). *Proc. Natl Acad. Sci. USA*, **82**, 968.

FRANÇOIS, J.C., SAISON-BEHMOARAS. T., CHASSIGNOL, M., THUONG, N.T., SUN, J.S. & HÉLÈNE, C. (1988*a*). Periodic cleavage of poly(dA) by oligothymidylates covalently linked to 1,10-phenanthroline-copper complex. *Biochemistry*, **27**, 2272.

FRANÇOIS, J.C., SAISON-BEHMOARAS, T. & HÉLÈNE, C. (1988*b*). Sequence-specific recognition of the major groove of DNA by oligodeoxynucleotides via triple helix formation. Footprinting studies. *Nucleic Acids Res.*, **24**, 11431.

FRANÇOIS, J.C., SAISON-BEHMOARAS, T., CHASSIGNOL, M., THUONG, N.T. & HÉLÈNE, C. (1988*c*). Nucléases artificielles: coupures spécifiques de la double hélice d'ADN 'ar des oligonucléotides liés au complexe cuivre-phénanthroline. *C.R. Acad. Sci. Paris*, **307** (série III), 849.

GAGNOR, C., BERTRAND J.R., THENET, S. and 7 others (1987). $\alpha$-DNA VI: comparative study of $\alpha$- and $\beta$-anomeric oligodeoxyribonucleotides in hybridization to mRNA and in cell free translation inhibition., *Nucleic Acids Res.*, **15**, 10419.

GAMPER, H.B., CIMINO, G.D., & HEARST, J.E. (1987). Solution hybridization of crosslinkable DNA oligonucleotides to bacteriophage M13 DNA. Effect of secondary structure on hybridization kinetics and equilibria. *J. Mol. Biol.*, **197**, 349.

HAREL-BELLAN, A., FERRIS, D.K., VINOCOUR, M., HOLT, J.T. & FARRAR, W.L. (1988). Specific inhibtion of c-*myc* protein biosynthesis using an antisense synthetic deoxyoligonucleotide in human T lymphocytes. *J. Immunol.*, **140**, 2431.

HEIKKILA, R., SCHWAB, G., WICKSTROM, E. and 4 others. (1987). A c-*myc* antisense oligodeoxynucleotide inhibits entry into S phase but not progress from G0 to G1. *Nature* **328**, 445.

HÉLÈNE, C., MONTENAY-GARESTIER, T., SAISON, T. and 7 others (1985). Oligonucleotides covalently linked to intercalating agents. A new class of gene regulatory substances. *Biochimie*, **67**, 777.

HÉLÈNE, C. & THUONG, N.T. (1988). Oligo-[$\alpha$]-deoxyribonucleotide covalently linked to intercalating agents. A new family of sequence-specific nucleic acid reagents. In *Nucleic Acids and Molecular Biology, Vol. 2*, Eckstein, F. & Lilley, D. (eds) p. 105. Springer-Verlag: Berlin.

HOLT, J.T., REDNER, R.L. & NIENHUIS, A.W. (1988). An oligomer complementary to c-*myc* mRNA inhibits proliferation of HL-60 promyeloctytic cells and induces differentiation. *Mol. Cell Biol.*, **8**, 963.

KEAN, J.M., MURAKAMI, A., BLAKE, K.R., CUSHMAN, C.D. & MILLER, P.S. (1988). Photochemical cross-linking of psoralen-derivatized oligonucleoside methylphosphonates to rabbit globin messenger RNA. *Biochemistry*, **27**, 9113.

KNORRE, D.G. & VLASSOV, V.V. (1985). Complementary addressed (sequence-specific) modification of nucleic acids. *Prog. Nucleic Acid Res. Mol. Biol.*, **32**, 291.

LE DOAN, T., PERROUAULT, L., CHASSIGNOL, M., THUONG, N.T. & HÉLÈNE, C. (1987*a*). Sequence-targeted chemical modifications of nucleic acids by complementary oligonucleotides covalently linked to porphyrins. *Nucleic Acid Res.*, **15**, 8643.

LE DOAN,, T., PERROUAULT, L., HÉLÈNE, C., CHASSIGNOL, M. & THUONG, N.T. (1986). Targeted cleavage of polynucleotides by complementary oligonucleotides covalently linked to iron-porphyrins. *Biochemistry*, **25**, 6736.

LE DOAN, T., PERROUAULT, L., PRASEUTH, D. and 5 others (1987*b*). Sequence-specific recognition, photocrosslinking and cleavage of the DNA double helix by an oligo-[α]-thymidylate covalently linked to an azidoproflavine derivative. *Nucleic Acid Res.*, **15**, 7749.

LEE, B.L., MURAKAMI, A., BLAKE, K., LIN, S.B. & MILLER, P.S. (1988). Intercalation of psoralen-derivatized oligodeoxyribonucleoside methylphosphonates with single-stranded DNA. *Biochemistry*, **27** 3197.

LEMAÎTRE, M., BAYARD, B. & LEBLEU. B. (1987). Specific antiviral activity of a poly(L-lysine)-conjugated oligodeoxyribonucleotide sequence complementary to vesicular stomatis virus N protein mRNA initiation site. *Proc. Natl Acad. Sci. USA*, **84**, 648.

MARCUS-SEKURA, C.J., WOERNER, A.M., SHINOZUKA, K., ZON, G. & QUINNAN. G.V. JR. (1987). Comparative inhibition of chloramphenicol acetyltransferase gene expression by antisense oligonucleotide analogues having alkyl phosphotriester, methylphosphonate and phosphorothioate linkages. *Nucleic Acids Res.*, **15** 5749.

MORVAN, F., RAYNER, B., IMBACH, J.L. and 5 others (1987). α-DNA II. Synthesis of unnatural α-anomeric oligodeoxyribonucleotides containing the four usual bases and study of their substrate activities for nucleases. *Nucleic Acids Res.*, **15**, 3421.

MOSER, H.E. & DERVAN, P.B. (1987). Sequence-specific cleavage of double helical DNA by triple helix formation. *Science*, **238**, 645.

PRASEUTH, D., LE DOAN, T., CHASSIGNOL, M. and 5 others (1988*b*). Sequence-targeted photosensitized reactions in nucleic acids by oligo-[α]-deoxynucleotides and oligo-[β]-deoxynucleotiodes covalently linked to proflavine. *Biochemistry*, **27**, 3031.

PRASEUTH, D., PERROUALT, L., LE DOAN, T., CHASSIGNOL, M., THUONG. N.T. & HÉLÈNE, C. (1988*a*). Sequence-specific binding and photocrosslinking of α and β oligodeoxynucleotides to the major groove of DNA via triple-helix formation. *Proc. Natl Acad. Sci. USA*, **85**, 1349.

STEIN, C.A., SUBASINGHE, C., SHINOZUKA, K. & COHEN, J.S. (1988). Physicochemical properties of phosphorothioate oligodeoxynucleotides. *Nucleic Acids Res.*, **16**, 3209.

SUN, J.S., ASSELINE, U., ROUZAUD, D., MONTENAY-GARESTIER, T., THUONG, N.T. & HÉLÈNE, C. (1987). Oligo-[α]-deoxynucleotides covalently linked to an intercalating agent. Double helices with parallel strands are formed with complementary oligo-[β]-deoxynucleotides. *Nucleic Acid Res.*, **15**, 6149.

SUN, J.S., FRANÇOIS, J.C., LAVERY, R. and 4 others (1988). Sequence-targeted cleavage of nucleic acids by oligo-[α]-thymidylate-phenanthroline conjugates. *Biochemistry*, **27**, 6039.

THUONG, N.T., ASSELINE, U., ROIG, V., TAKASUGI, M. & HÉLÈNE, C. (1987). Oligo(α-deoxynucleotides) covalently linked to intercalating agents: differential binding to ribo- and deoxyribopolynucleotides and stability towards nuclease digestion. *Proc. Natl Acad Sci. USA*, **84**, 5129.

TOULMÉ, J.J., KRISCH, M.M., LOREAU, N., THUYONG, N.T. & HÉLÈNE, C. (1986). Specific inhibition of mRNA translation by complementary oligonucleotides covalently linked to intercalating agents. *Proc. Natl Acad. Sci. USA*, **83**, 1227.

VERSPIEREN, P., CORNELISSEN, A.W.C.A., THUONG, N.T., HÉLÈNE, C. & TOULMÉ, J.J. (1987). An acridine-linked oligodeoxynucleotide targeted to the common 5′ end of trypanosome mRNAs kills cultured parasites. *Gene*, **61**, 307.

WICKSTRÖM, E.L., BACON, T.A., GONZALEZ, A., FREEMAN, D.L., LYMAN, G.H. & WICKSTRÖM, E. (1988). Human promyelocytic leukemia HL-60 cell proliferation and c-*myc* protein expression are inhibited in an antisense pentadecadeoxynucleotide targeted against c-*myc* mRNA. *Proc. Natl. Acad. Sci. USA*, **85**, 1028.

ZÉRIAL, A., THUONG, N.T. & HÉLÈNE, C. (1987). Selective inhibition of the cytopathic effect of type A influenza viruses by oligodeoxynucleotides covalently linked to an intercalating agent. *Nucleic Acids Res.*, **15**, 9909.